# Feasibility of self-administered dried blood spot collection for cardiometabolic profile analysis in a population-based sample of young adults

**DOI:** 10.1371/journal.pone.0334023

**Published:** 2025-10-08

**Authors:** Katherine M. Livingstone, Kathleen M. Dullaghan, Jeffrey M. Craig, Barbara Brayner, Sarah A. McNaughton

**Affiliations:** 1 Institute for Physical Activity and Nutrition, School of Exercise and Nutrition Sciences, Deakin University, Geelong, Victoria, Australia; 2 Murdoch Children’s Research Institute, Royal Children’s Hospital, Parkville, Victoria, Australia; 3 Institute for Mental and Physical Health and Clinical Translation, School of Medicine, Deakin University, Geelong, Victoria, Australia; 4 Health and Well-Being Centre for Research Innovation, School of Human Movement and Nutrition Sciences, University of Queensland, St Lucia, Queensland, Australia; 5 School of Exercise and Nutrition Sciences, Deakin University, Geelong, Victoria, Australia; Saga University, JAPAN

## Abstract

Young adults may have poor cardiometabolic health that is undetected due to infrequent health checks. Feasibility of self-administration of blood samples using dried blood spot (DBS) cards for remote screening of this population group is unknown. The primary aim of this study was to examine the feasibility of collecting self-administered blood samples using DBS cards for cardiometabolic profile analysis in a population-based sample of young adults. Between April and November 2022, Australian young adults (18–30 years) completed an online survey and were mailed a self-administered DBS collection kit. Participants completed an open-ended question on any difficulties with the blood collection process. Samples were returned for an 8-item cardiometabolic profile analysis. Of the 506 participants mailed a collection kit, 72% (n = 366; mean 23.5 [SD 3.9] years; 53% female) returned their samples. Of mailed collection kits, 46% (n = 235 of 506) of participants returned samples that were adequate for all eight cardiometabolic profile measures. The participants who reported difficulties with the process (63%; n = 313) were followed up twice, on average (range 1–12 times), by the researcher to provide support, of which n = 155 proceeded to provide an adequate sample for all eight cardiometabolic measures. A lack of blood (75%; n = 235) was the most reported difficulty with sample collection. With the provision of support, self-administered dried blood spot collection for cardiometabolic profile analysis shows promise in a population-based sample of young adults.

## Introduction

Poor cardiometabolic health is a strong predictor of cardiovascular disease (CVD) and early death globally [1]. In high income countries, such as Australia, non-communicable diseases remain the major cause of mortality, accounting for over 90% of all deaths, with ischemic heart disease and stroke among the leading causes of years of lives lost [2]. Early detection of poor cardiometabolic health, such as high levels of HbA1C and triglycerides and low levels of HDL-cholesterol, may help reduce the global burden of death from non-communicable diseases.

Young adulthood, broadly defined as 18–30 years of age, is often characterised by high risk behaviours that negatively impact on cardiometabolic health [3]. This includes low intakes of fruits and vegetables and high intake of discretionary foods and alcoholic beverages, contributing to high rates of overweight and obesity [4,5]. Estimates from the 2011–2018 US national surveys suggest that up to 38% of young adults (18–29 years) had dyslipidemia and up to 21% had two or more cardiometabolic diseases [6]. In Australia, as many as 55% of young adults (18–34 years) were reported to have dyslipidemia in 2011–2012 [7].

Most CVD guidelines focus on risk assessments in middle-aged and older adults [7,8], missing early transitions in cardiometabolic health that may represent critical windows of opportunity for averting the development of CVD. In 2018, young Australians (15–24 years) report visiting their general practitioners the least often of all age groups (75% vs 98% of adults aged 85 years and older) [9], which may lead to poor cardiometabolic health in young adults being undetected. The prevalence of poor cardiometabolic health is likely to increase with the rising cost of living, where the cost of healthy foods has risen more than that of unhealthy foods [10].

Dried blood spot (DBS) card collection kits are being increasingly used for remote screening of health conditions in Australia and internationally [11,12], including in hard-to-reach and low-income groups [13]. DBS technology is an alternative to venous blood collection performed by a trained professional and involves obtaining capillary blood by pricking the finger with a safety lancet and blotting the blood onto filter paper. As samples can be self-collected and stored at room temperature, this method is likely to significantly reduce personnel, storage, and shipping costs, while simultaneously improving reach, convenience, and accessibility through greater autonomy, privacy and cultural safety [13]. Assays have been developed and validated against serum samples for a wide range of analytes in DBS cards [14], including cardiometabolic measures such as high sensitivity C-reactive protein (hsCRP), triglycerides and insulin [15,16]. However, research on the feasibility of self-collection of DBS samples in population-based studies is limited, with large variations in response rates (26% to 71%) and over-representation of middle or older age adults and clinical populations [17–19]. Moreover, to our knowledge, no studies have investigated the feasibility of collecting DBS for cardiometabolic profile analysis in this age group and determined their corresponding risk of poor cardiometabolic health. The simultaneous investigation of blood sugar levels, using haemoglobin A1C (HbA1C), and blood lipid levels, including HDL-cholesterol, provides a holistic analysis of cardiometabolic profile. Therefore, the primary aim of this study was to determine the feasibility of collecting self-administered blood samples using DBS cards for cardiometabolic profile analysis in a population-based sample of young adults. The secondary aim was to identify the prevalence of young adults with at-risk profiles for these cardiometabolic markers.

## Materials and methods

### Study design

Participants were recruited between 21 April 2022 and 21 November 2022 into the MyMeals study, an online survey and self-administered DBS collection study to understand food choices and cardiometabolic health in young adults. Information is reported according to the STROBE-nut checklist for observational studies (S1 Table) [20]. Procedures align with the Declaration of Helsinki, and the study was approved by the Deakin University Human Ethics Committee (reference number 2021_182). Participants provided informed consent.

### Participants

Young adults (aged 18–30 years) were recruited using paid and targeted advertisements on Facebook and Instagram. Although a convenience sample recruitment approach was used, social media advertisements were used to boost the representation by age and sex. Participants were considered eligible if: i) they lived in Australia; ii) they were not following a vegan or vegetarian diet (which would limit generalisability of food choice data to the wider young adult population in the MyMeals study); iii) they were not currently pregnant or breastfeeding, iv) English was the primary language spoken at home and v) they consented to provide finger-prick blood spot samples while in a fasted state using a kit mailed to their home.

### Study procedures

Social media advertisements directed interested individuals to the survey via a study-specific link hosted by Qualtrics Survey Software (Qualtrics, Provo, UT). Potential participants were directed to an online plain language statement, screening questions, and online consent statement. Participants meeting the inclusion criteria provided written consent by indicating agreement to the online consent statement (tick box). Participation was voluntary, and participants could withdraw at any time. Consenting and eligible participants were then contacted by the research team to arrange the mailout of their DBS collection kit and access to the online survey. To minimise order bias, participants were randomised to complete the DBS sample collection before or after the online survey. Participants were instructed to return their DBS samples to NutriPATH Pathology (Ashburton, Victoria) via an Express Post padded envelope provided in their collection kit.

A researcher (KMD) monitored access to the online survey to determine whether participants had any difficulties collecting their sample, and contacted participants to provide additional support if they did not progress past the DBS collection instructions in the survey or if difficulties were reported. Participants were also encouraged to contact the research team if they had followed all instructions and still experienced difficulties. Participants who did not access the online survey or return their blood spot samples were contacted by the research team via electronic text message (or via email if no mobile number was provided) two weeks after the DBS collection kit mailing date to ensure all materials had been received. Two subsequent text messages and a phone call, over a six-week period, were used to reach participants prior to deeming participants lost to follow up. Participants received an AUD30 e-voucher via email once both the online survey had been completed and their DBS sample had been received by the pathology company.

### Blood spot collection kit

Participants were mailed out a DBS collection kit prepared by NutriPATH Pathology (Ashburton, Victoria) in consultation with the research team. The kit contained a request form, collection instructions, sample transport bag, welcome sheet/checklist, two collection cards labelled 1 and 2, sterile alcohol wipes, two safety lancets (Sarstedt Safety lancet Extra Ø needle (85.1017) or BD Microtainer contact-activated lancet (366578)), two adhesive bandages, and an Express Post padded envelope. In response to feedback from participants, the type and number of lancets provided changed part way through the study, where the number increased to three, then four lancets, and the type changed to BD Microtainer contact-activated lancet (366578). Standard collection instructions were adapted by the research team for the purpose of the MyMeals study. Participants were instructed to collect samples in a fasted state (for at least 8 hours) and to allow the sample to air dry for at least 3 hours prior to packaging for posting. Information on date and time of collection and hours fasted was collected on the request form. Detailed written step-by-step instructions were provided, including images of sample collection at each step (S1 Fig), a link to a 4-minute YouTube video (not study specific and provided by NutriPATH Pathology; https://www.youtube.com/watch?v=ts97s227cww) demonstrating how to collect a sample, and an FAQ page specifically designed for this study with information about the study procedures and tips for getting a good sample. For example, participants were encouraged to run their hands under warm water for 30 seconds to improve blood flow prior to collection of the sample. Participants were instructed to complete card 1 first, followed by card 2. Collection card 1 was a 12-spot card used to run a standard 8-item cardiometabolic profile panel provided by NutriPATH Pathology. Card 2 was a 5-spot Whatman^TM^ 903 protein saver card (Sigma-Aldrich, Milan, Italy) retained by the research team for future testing.

All participants who returned their sample were contacted via email by a member of the research team (KMD). When adequate samples were returned, participants were provided with personalised information on standard blood biomarkers that would be routinely screened for as part of a health practitioner screening (i.e., fasting cholesterol, glucose and insulin results), which indicated if they were within normal references ranges or at risk for any of the markers. Participants identified at risk for any cardiometabolic marker (i.e., outside of reference ranges) were encouraged to discuss their results with their medical practitioner. When the samples were inadequate for analysis of any markers, participants were informed that it was not possible to provide information on their cardiometabolic profile.

### Biochemical assays

The 12-spot DBS cards were shipped by NutriPATH Pathology to ZRT Laboratory (Beaverton, OR) for cardiometabolic profile analysis. The cardiometabolic panel included assessment of total cholesterol (mg/dL), HDL-cholesterol (mg/dL), LDL-cholesterol (mg/dL), VLDL-cholesterol (mg/dL), hsCRP (mg/L), triglycerides (mg/dL), HbA1C (%) and insulin (µIU/mL). Data from ZRT Laboratory demonstrate that blood spot samples show a high correlation with venous serum samples (hsCRP, *r* = 0.99; fasting insulin, *r* = 0.93; fasting triglycerides, *r* = 0.95) and analyte stability in dried blood [16]. Each circle on the collection cards was 15 mm in diameter (equivalent to up to 80 µL of blood) to enable up to four 6.0 mm punches for analysis, with a different number of punches required depending on the assay. For example, three 6.0 mm punches were required for enzymatic assays for triglycerides, whereas insulin required two 6.0 mm punches for use in commercial ELISA kits.

Details on preparation procedures and assays are provided elsewhere [15]. Briefly, for triglycerides, two 6.0 mm blood spot disks were punched into deep 96-well plates using an automated blood spot puncher (Wallac MultiPuncher). Triglycerides were extracted from the dried blood spot samples with 200 μL of methanol for 2 hours at 37°C. Triglyceride levels were determined using the assay kit as described by the manufacturer (Randox Laboratories Ltd). For hsCRP, one 6.0 mm blood spot disk was punched into the 96-well plate and was extracted with 200 μL of extraction buffer for 2 hours on a microtiter plate shaker at room temperature. Following serial dilution of the standard to prepare a standard curve, a 20 μL aliquot was added to the 96-well assay plate followed by 100 μL of the CRP enzyme conjugate from the assay kit (DRG International, Inc). hsCRP levels were determined using the assay kit as described by the manufacturer. For insulin, two 6.0 mm blood spot disks were punched into the 96-well plates. The dried blood spot samples were extracted with 350 μL of extraction buffer for 90 minutes on a microtiter plate shaker at room temperature. Rehydrated blood spots were tested using commercial ELISA kits from DRG. Bio-Rad Lypocheck Immunoassay Plus control levels 1, 2, and 3 were used for establishing controls and calibrators for the blood spot insulin. Lipid control levels 2 and 3 from Randox were used for triglycerides, and wide-range calibrators from Core Laboratory Supplies, Inc. were used for hsCRP. Further details are available elsewhere [15,16].

### Cardiometabolic risk

Age- and sex-appropriate reference ranges defined by ZRT Laboratory were used to determine whether samples were within range (including optimal if applicable): insulin (1–15 µIU/mL; optimal 2–6 µIU/mL); hsCRP (<3 mg/L); HbA1c (<6%; optimal 3.5–5.5%); triglycerides (<150 mg/dL); total cholesterol (<200 mg/dL); HDL-cholesterol (≥40 mg/dL); LDL-cholesterol (<130 mg/dL; optimal <100 mg/dL) and VLDL-cholesterol (<30 mg/dL). If the DBS card did not contain adequate blood for analysis of all cardiometabolic markers, then markers were prioritised for analysis in the following order: HbA1C, HDL-cholesterol, total cholesterol, triglycerides, VLDL-cholesterol, LDL-cholesterol, hsCRP, insulin.

### Online survey

The online survey was delivered via Qualtrics Survey Software (Qualtrics) and included 9 items on demographic characteristics (age, sex, relationship status, country of birth, current postcode, highest educational attainment, individual weekly income, living arrangements) and 23 items on health behaviours (diet, physical activity, smoking status, height, weight, sleep duration and self-reported perception of health). Two survey items assessed feasibility of collecting DBS samples: directly following sample collection, participants were asked whether they perceived they had any difficulties completing the DBS card collection (yes/no). If the response was ‘yes’, they were presented with an open-ended text box to explain why.

Information on demographic and health questionnaire items is described elsewhere [21]. Briefly, body mass index (BMI) was estimated from self-reported weight (kg) and height (m). Participants were categorised into underweight/normal weight (BMI < 25 kg/m^2^), overweight (BMI ≥ 25 and <30 kg/m^2^) and obesity (BMI ≥ 30 kg/m^2^). Physical activity was assessed using the 4-item short International Physical Activity Questionnaire [22]. Minutes of activity per week were used to categorise participants according to whether or not they met the physical activity guidelines of at least 150 minutes of moderate to vigorous activity per week [23]. Number of hours of sleep on a week day was used to categorise participants into whether they met the sleep duration guidelines of between 7–9 hours per night [24]. Information on dietary intakes was collected using 17 previously tested questionnaire items [25–27]. Data were collected on intakes of fruit (serves/day), vegetables (serves/day), bread (serves/day), rice, pasta or noodles (frequency consumed per month), meat and alternatives (frequency consumed per month), milk, cheese and yoghurt (frequency consumed per month), discretionary foods and drinks and alcoholic beverages (frequency consumed per month) [28]. Responses for frequency per month were converted to daily serve equivalents [29]. Dietary items were used to derive the 2013 Australian Dietary Guideline Index (DGI), a food-based overall diet quality index [21,29–33]. As detailed elsewhere [21], 10 food components were included in the DGI estimation, with items scored out of 10 (0 indicating the guideline was not met). Overall DGI scores ranged between 0 and 100, with a higher score indicating better diet quality.

### Statistical analysis

Descriptive statistics were used for continuous variables (mean and standard deviation, SD) and categorical variables (frequency). Histograms were visually inspected for normality; median and interquartile range (IQR) were reported for non-normally distributed variables. The percentage of returned DBS samples was calculated as the number of participants who returned a sample with adequate blood to estimate at least one cardiometabolic measure divided by the number of participants who were sent DBS kits. Chi-squared tests (categorical variables) and t tests (continuous variables) were used to compare participant characteristics between those who returned an adequate sample and those who did not return their collection cards, and to compare the number of participants who provided an adequate sample for all eight cardiometabolic measures according to whether participants reported any difficulties collecting samples, with p < 0.05 used to determine statistical significance. Two-way plots were used to graphically present each cardiometabolic marker for participants who were identified as having a “normal” profile and an “at-risk” profile based on the reference ranges described earlier. STATA software (StataCorp LLC, College Station, TX, version 17) was used for statistical analyses. Open ended responses for reasons for difficulties collecting DBS samples were coded by one researcher (KML) using conventional content analysis. Key themes were identified and discussed with a second researcher (KMD) and summarised, with example quotes extracted.

## Results

### Feasibility of sample collection

**Fig 1:**
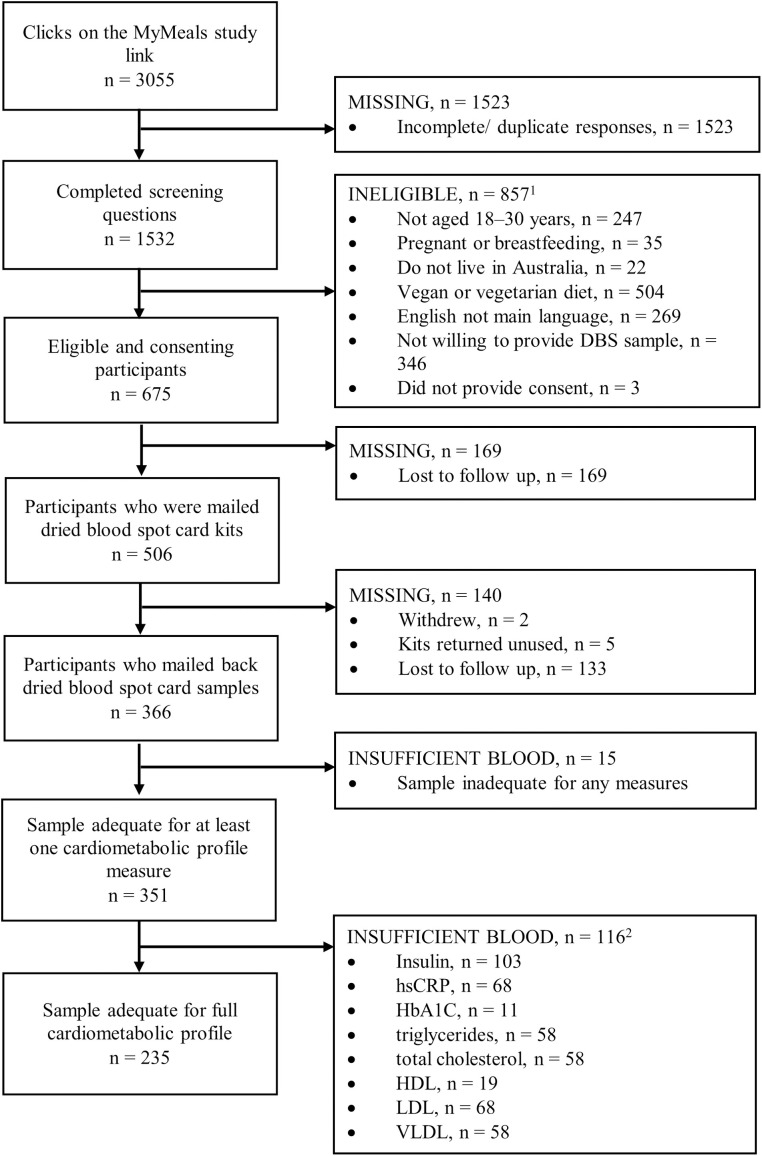
Flow diagram of participants included in the MyMeals study dried blood spot card collection.

**Fig 2:**
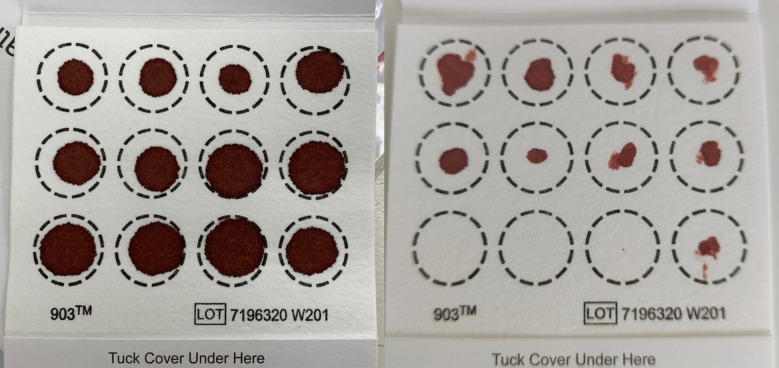
Participant dried blood spot card collection examples of A) a ‘good quality’ sample that was adequate for analysis of all eight cardiometabolic profile markers and B) a ‘poor quality’ sample that was adequate for analysis of only two cardiometabolic profile markers.

As shown in Fig 1, the MyMeals Study link was accessed 3055 times. A total of 779 individuals completed the screening questions; of these, 675 participants were eligible, provided consent and commenced the online survey. A total of 169 participants were lost to follow up due to not confirming their postal address for dispatch of the DBS kit. A total of 506 participants were sent a DBS kit, of which five participants were excluded due to kits returned to sender and two participants withdrew from the study. A further 133 participants were lost to follow up due to non-completion of the online survey and non-return of the collection cards. A total of 366 participants posted their DBS samples to the pathology company for analysis. This resulted in 54% of eligible participants returning a sample, and a response rate of 72% for those who were sent a DBS kit. Of the mailed kits, 69% (n = 351) were adequate for at least one cardiometabolic profile measure, 68% (n = 342), 56% (n = 285) and 56% (n = 283) were adequate for at least two, four and six measures, respectively, while 46% (n = 235) of samples were adequate for all eight cardiometabolic profile measures. Fig 2 shows an example of a DBS sample that was adequate for measuring all eight cardiometabolic markers and a DBS sample that was adequate for two markers only. A comparison of response rates and adequacy of samples according to lancet number and type is provided in S2 Table.

Numbers are not additive as some participants were excluded for multiple reasons.

1, Each circle was 15 mm in diameter (equivalent to up to 80 µL of blood) and would enable up to fourteen 3.2 mm punches for analysis;

2, Samples were not adequate for analysis of all eight cardiometabolic measures; analysis priority was HbA1C, HDL, total cholesterol, triglycerides, VLDL, LDL hsCRP, Insulin.

**Fig 3:**
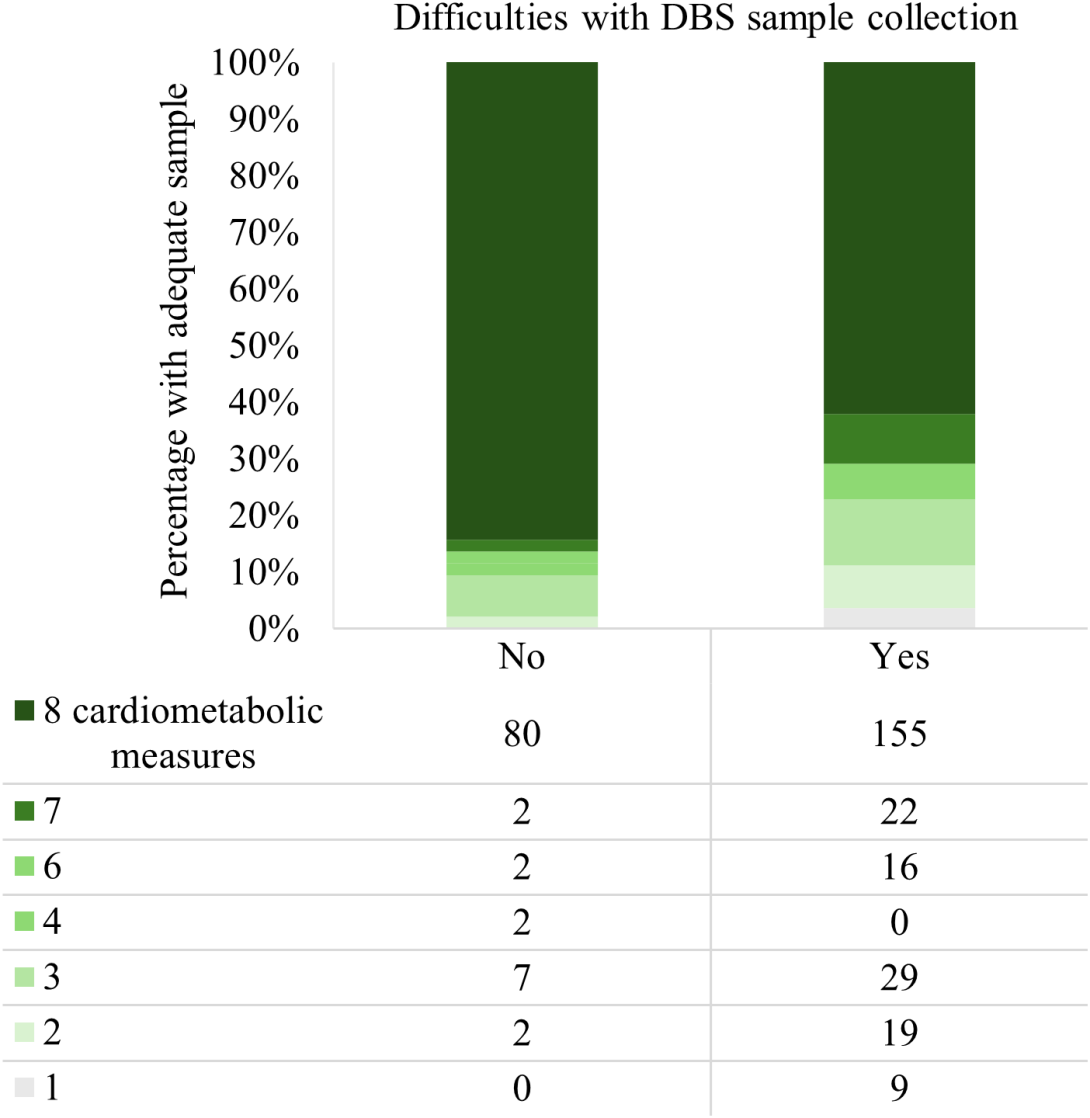
Number of successful cardiometabolic measures according to participant difficulties with sample collection.

A total of 188 participants received a reminder (via SMS, or via email if no mobile number was provided) to complete their online survey and DBS sample collection, 119 were sent a second reminder, there were 110 follow up attempts by phone (or email), and 84 participants received a third reminder. Of these, 77 were deemed lost to follow up after no reply to the third reminder. A total of 289 participants (57%) indicated in the online survey that they experienced difficulties with sample collection, and an additional 24 participants only reported difficulties after being contacted by the researcher (KMD) as part of a scheduled follow up. These 313 participants were contacted twice, on average (range 1–12 times) by the researcher to provide support. Of the 351 participants who returned a sample adequate for at least one cardiometabolic marker, 71% reported difficulties. More participants who did not report any difficulties provided an adequate sample for all eight cardiometabolic measures, compared with participants who did report difficulties (79% vs 62%; p = 0.002; Fig 3).

**Fig 4:**
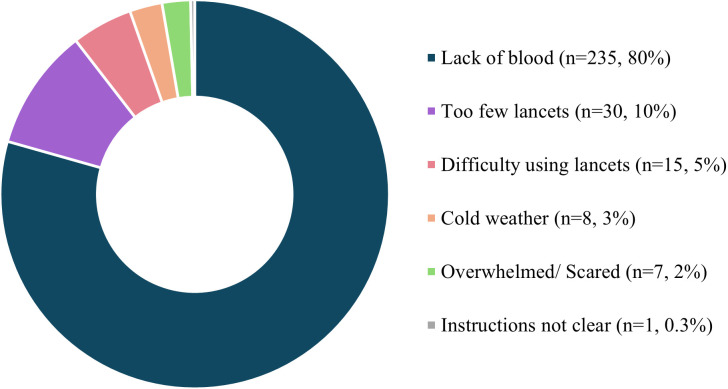
Summary of dried blood spot sample collection difficulties reported by participants.

Of the participants who mailed back their samples (n = 366), 72% (n = 262) reported difficulties, while of the participants who did not mail back their DBS samples (n = 133), 38% (n = 51) reported difficulties. As shown in Fig 4, the main difficulties experienced included lack of blood (n = 235), too few lancets (n = 30), difficulty using lancets (n = 15), cold weather (n = 8), feeling overwhelmed/ scared (n = 7) and unclear instructions (n = 1). Table 1 provides quotes from participants to provide additional context to the main difficulties identified.

**Table 1:** Main reasons for difficulties completing dried blood spot card sample collection.

Theme	Example quotes
Lack of blood	“Difficulties getting enough blood; had to prick multiple fingers.” (Participant 234, Female, 23 years)
	“Even though I danced to two songs before to get my heart rate up and ran my hand under hot water I experienced blood flow issues. I used three lancets on three different fingers (the last one I used on my right hand and that went better).” (Participant 202, Female, 19 years)
	“After 4 spots I was struggling to get blood out of my fingers. I drank some more water and waited a few minutes then tried again with another finger and it was a lot better.” (Participant 22, Female, 18 years)
	“Blood flow was really difficult on card 1. A few good samples, but some look like the low-quality ones. Sorry!” (Participant 44, Male, 23 years).
	“Difficulties with completing card 1 with any ‘good’ samples. Referred to FAQ on tips to improve circulation, video and attempted with 3 different fingers without success.” (Participant 19, Male, 26 years).
Too few lancets	“I couldn’t produce much blood from each finger prick and wasn’t provided enough lancets to finish the sample. I was given 4 and would probably have needed approx. 12.” (Participant 166, Male, 25 years).
Difficulty using lancets	“The lancets didn’t puncture very well.” (Participant 11, Male, 24 years).
	“One lancet did not lead to any blood flow and the other three only led to a small amount of flow.” (Participant 7, Male, 20 years)
	“The lancets didn’t puncture very well and the blood didn’t drop even though I kept massaging the area.” (Participant 55, Female, 24 years).
Cold weather	“I couldn’t seem to get enough blood from my fingers - it’s rather cold this morning.” (Participant 172, Female, 29 years).
	“Not enough blood as weather was cold.” (Participant 133, Male, 29 years).
	“Because it was so cold, I struggled to get good blood spots. I used all the lancets and have filled all the bubbles, but each bubble still has a lot of white space around the edge.” (Participant 188, Female, 28 years).
Overwhelmed/ scared	“Had trouble getting enough blood out of my finger and felt overwhelmed by sight of blood so was unable to complete the sample collection” (Participant 24, Male, 18 years) “I was scared and needed someone else to prick me because of the anticipation stopping me from doing it myself” (Participant 24, Female, 21 years)

### Participant characteristics according to sample return

Table 2 shows the demographic characteristics of the participants according to those who did not return their collection cards, those who returned adequate samples for one to seven cardiometabolic measures and those who returned adequate samples for all eight measures. Of participants who returned an adequate sample for all eight measures, mean age was 23.8 (3.90) years; 49% were female, 83% were born in Australia, 56% had a high education status, 45% had a high income, 66% lived in an area of low socio-economic disadvantage, 52% were in a relationship, 66% and 78% met physical activity and sleep guidelines, respectively. Mean BMI was 26.5 (7.1) kg/m^2^, mean DGI was 64.1 (8.6), 7% were current smokers, 80% reported excellent to good health and 46% had a history of heart disease or diabetes among participants who returned an adequate sample. More participants who did not return their collection cards had a low education status and were current smokers compared to participants who returned a sample that was adequate for one to seven cardiometabolic measures (62.5% vs 38.8%; p = 0.033 and 16.7% vs 2.6%; p = 0.004 respectively). No other significant differences in participant characteristics were noted (Table 2).

**Table 2:** Participant characteristics who did not return their dried blood spot collection cards (i.e., lost to follow up) compared to participants who returned a sample that was adequate for cardiometabolic profile measures to be analysed.

Characteristic	No collection card returned (n = 133)^1^	Returned adequate sample			
		1–7 measures (n = 116)	p-value^2^	All 8 measures (n = 235)	p-value^2^
Age (years), Mean ± SD	23.0 (3.94)	23.5 (3.90)	0.79	23.8 (3.9)	0.14
Sex, n (%)			0.06		0.43
Female	71 (46.2)	47 (41.6)		113 (48.9)	
Male	61 (53.8)	66 (58.4)		118 (51.1)	
Country of birth, n (%)			0.78		0.18
Australia	18 (75.0)	90 (77.6)		196 (83.4)	
Other	6 (25.0)	26 (22.4)		39 (16.6)	
Education, n (%)			**0.033**		0.43
Low	15 (62.5)	45 (38.8)		104 (44.3)	
High	9 (37.5)	71 (61.2)		131 (55.7)	
Income, n (%)			0.34		0.64
Low	15 (62.5)	60 (51.7)		129 (54.9)	
High	9 (37.5)	56 (48.3)		106 (45.1)	
SEIFA, n (%)			0.12		0.96
Low (most disadvantaged)	12 (52.2)	40 (34.8)		78 (33.6)	
High	11 (47.8)	75 (65.2)		154 (66.4)	
Relationship status, n (%)			0.38		0.53
In a relationship	9 (37.5)	55 (47.4)		122 (51.9)	
Single	15 (62.5)	61 (52.6)		113 (48.1)	
BMI (kg/m^2^), Mean ± SD	25.9 (7.4)	25.2 (6.5)	0.70	26.5 (7.1)	0.11
Dietary Guideline Index, Mean ± SD	64.3 (7.71)	64.7 (8.5)	0.86	64.1 (8.7)	0.48
Smoking status, n (%)			**0.004**		0.06
Never smoked/ Ex-smoker	20 (83.3)	113 (97.4)		219 (93.2)	
Current smoker	4 (16.7)	3 (2.6)		16 (6.8)	
Physical activity, n (%)			0.28		0.52
Do not meet guidelines	5 (20.8)	37 (31.6)		80 (68.4)	
Meet guidelines	19 (79.2)	79 (33.8)		155 (66.2)	
Sleep duration, n (%)			0.63		0.27
Do not meet guidelines	8 (33.3)	33 (28.5)		53 (22.6)	
Meet guidelines	16 (66.7)	276 (71.6)		182 (77.5)	
Self-reported health, n (%)			0.08		0.94
Excellent or good	15 (62.5)	92 (79.3)		187 (79.6)	
Fair or poor	9 (37.5)	24 (20.7)		48 (20.4)	
Family history of heart disease/diabetes			0.63		0.31
No	13 (54.2)	69 (59.5)		128 (54.5)	
Yes	11 (45.8)	47 (40.5)		107 (45.5)	

1, Sample varies due to missing data for demographic characteristics, 2, P values represent t test (continuous variables) or chi squared tests (categorical; variables) to examine statistical differences between participant characteristics for those who did not return their collection card and those who returned their collection card; SEIFA, Socio-Economic Indexes for Areas using the Index of Relative Socio-economic Disadvantage; Education: low (no formal qualifications, year 10 or equivalent, year 12 or equivalent, trade/apprenticeship, certificate/diploma) and high (University degree, higher University degree); Income: low (AUD0–999/week) or high (>AUD1000/week); SEIFA: area-level disadvantage was divided into deciles ranging from the most disadvantaged (decile 1) to least disadvantaged (i.e., most affluent—decile 10): Low (deciles 1–5) and High (deciles 6–10); Physical activity: ≥ 150 min of moderate or vigorous activity/week; Sleep: 7–9 hours/night.

### Cardiometabolic profile of returned samples

The cardiometabolic profile of adequate samples returned by participants overall and by sex is shown in Table 3. All participants reported being fasted, with most participants (65%) completing DBS collection between 7:00 AM and 10:30 AM. Overall, the concentration of total cholesterol was 187.6 (40.2) mg/dL, HDL-cholesterol was 39.7 (15.7) mg/dL, LDL-cholesterol was 128 (38.7) mg/dL and HbA1C was 4.94 (0.55) %, while median VLDL-cholesterol was 18 (10) mg/dL, insulin was 7.93 (5.01) µIU/mL and hsCRP was 0.60 (1.39) mg/L. Seventy four percent of participants (n = 260) were identified as at risk for at least one cardiometabolic marker (82% of males and 65% of females). More males than females were at risk for all lipid markers (total cholesterol: males 42% vs females 28%; HDL-cholesterol: males 66% vs females 39%; LDL-cholesterol: males 55% vs females 33%; VLDL-cholesterol: males 17% vs females 12%; triglycerides: males 16% vs females 11%), HbA1c (males 4% vs females 2%) and insulin (males 12% vs females 11%), while more females (19%) were at risk for hsCRP than males (9%). The cardiometabolic profile of participants according to whether they were normal (inside of the reference and optimal ranges) or at risk (outside of the reference and optimal ranges) are shown in S2 Fig and S3 Fig.

**Table 3:** Cardiometabolic profile of young adults overall and by sex.

Marker	Adequate sample^1^, n (%)	Overall	Males	Females
Total cholesterol (mg/dL)	293 (80.1)			
Concentration, mean (SD)		187.6 (40.1)	192.6 (42.1)	182.2 (37.3)
Optimal range, n (%)		–	–	–
Reference range (<200 mg/dL), n (%)		190 (64.9)	89 (58.2)	96 (71.6)
At risk (≥200 mg/dL), n (%)		103 (35.5)	64 (41.8)	38 (28.4)
Insufficient blood, n		73	36	36
HDL-cholesterol (mg/dL)	332 (90.7)			
Concentration, mean (SD)		39.7 (15.7)	36.6 (12.7)	43.5 (17.9)
Optimal range, n (%)		–	–	–
Reference range (≥40 mg/dL), n (%)		154 (46.4)	59 (34.5)	94 (60.7)
At risk (<40 mg/dL), n (%)		178 (53.6)	112 (65.5)	61 (39.4)
Insufficient blood, n		34	18	15
LDL-cholesterol (mg/dL)	283 (77.3)			
Concentration, mean (SD)		127.5 (38.7)	135.3 (38.4)	118.9 (37.5)
Optimal range (<100 mg/dL), n (%)		78 (27.8)	28 (19.3)	49 (37.1)
Reference range (<130 mg/dL), n (%)		159 (56.2)	66 (45.5)	89 (67.4)
At risk (≥130 mg/dL), n (%)		124 (44.0)	79 (54.5)	43 (32.6)
Insufficient blood, n		83	44	38
VLDL-cholesterol (mg/dL)	293 (80.1)			
Concentration, median (IQR)		18 (10)	18 (11)	18 (10)
Optimal range, n (%)		–	–	–
Reference range (<30 mg/dL), n (%)		250 (85.3)	127 (83.0)	118 (88.1)
At risk (≥30 mg/dL), n (%)		43 (14.7)	26 (17.0)	16 (11.9)
Insufficient blood, n		73	36	36
High sensitivity CRP (mg/L)	283 (77.3)			
Concentration, median (IQR)		0.60 (1.39)	0.50 (1.0)	0.70 (1.8)
Optimal range, n (%)		–	–	–
Reference range (<3 mg/L), n (%)		243 (85.9)	126 (90.7)	112 (81.2)
At risk (≥3 mg/L), n (%)		40 (14.1)	13 (9.4)	26 (18.8)
Insufficient blood, n		83	50	32
Triglycerides (mg/dL)	293 (80.1)			
Concentration, median (IQR)		90 (52)	90 (54)	91 (53)
Optimal range, n (%)		–	–	–
Reference range (<150 mg/dL), n (%)		252 (86.0)	128	119
At risk (≥150 mg/dL), n (%)		41 (14.0)	25 (16.3)	15 (11.2)
Insufficient blood, n		73	36	36
Haemoglobin A1C (HbA1C)	340 (92.9)			
Concentration, mean (SD)		4.94 (0.55)	5.04 (0.59)	4.82 (0.48)
Optimal range (3.5–5.5%), n (%)		299 (88.0)	146 (83.0)	147 (93.6)
Reference range (<6%), n (%)		329 (96.8)	169 (96.0)	154 (98.1)
At risk (≥6%), n (%)		11 (3.20)	7 (3.98)	3 (1.91)
Insufficient blood, n		26	13	13
Insulin (µIU/m)	248 (67.8)			
Concentration, median (IQR)		7.93 (5.01)	8.3 (4.8)	7.45 (4.6)
Optimal range (2–6 µIU/mL), n (%)		74 (29.8)	30 (24.6)	41 (33.6)
Reference range (1–15 µIU/mL), n (%)		223 (90.0)	108 (88.5)	111 (90.1)
At risk (>15 µIU/mL), n (%)		25 (10.0)	14 (11.5)	11 (9.02)
Insufficient blood, n		188	67	48

CRP, C-Reactive Protein; IQR, Interquartile range; 1, An adequate sample was one where the analysis of at least one cardiometabolic profile marker was possible. Missing data for sex (n = 8); Prefer not to say for sex (n = 8).

## Discussion

This study investigated the feasibility of self-administered dried blood spot collection in a population-based sample of young adults**.** Although many participants reported some difficulties completing sample collection, they were encouraged to return their samples regardless, leading to a high response rate (72%; n = 366 of 506). A total of 69% of DBS kits mailed (n = 351 of 506) were adequate for at least one cardiometabolic profile measure, and 46% (n = 235 of 506) were adequate for all eight cardiometabolic profile measures. Minimal differences in participant characteristics were identified between participants who returned an adequate blood sample and those who did not return their sample; however, there were trends to suggest that there was response bias with participants with a higher socio-economic position more likely to return samples. Collectively, these findings show that self-administered dried blood spot collection is feasible if step-by-step instructions and support are provided, and some targeted approaches may be necessary for different population groups to achieve adequate samples. Moreover, our secondary aim indicated high proportions of young adults identified as at risk of poor cardiometabolic health and highlights the need for screening in this at-risk population group.

The high response rate and adequacy of samples returned in this study is comparable to previous research [17,18]. In a population-based study of 4597 Norwegian women, 70% of DBS cards sent were mailed back and 93% were adequate for at least one biomarker analysis [18]. Similarly, in a population-based study of 257 US adults, 70% of DBS cards sent were mailed back; however, the authors report that of the eligible participants, only 26% agreed to participate and returned their sample [17]. These researchers observed that participants who were Black (compared to White), smoked, had a lower education and higher weight status were less likely to participate and return adequate samples [17,18], which is consistent with socio-economic patterning observed in the present findings. The feasibility of DBS self-collection may be higher in individuals with higher socio-economic position, regardless of whether a financial incentive was offered [17] or not [18]. However, the high response rate observed in the present study may be partially attributable to the personalised support provided to participants by the research team. In previous research [18], phone calls were received from 7% of participants, whereas in this study, the research team proactively followed up 62% of participants who were sent kits. Moreover, the support (step-by-step instructions, video, FAQs) was more comprehensive than in previous research, where simple step-by-step instructions were provided [18]. It is likely that without this support, the response rate would have been lower. However, comparison with previous research is challenging since the present study was in 18–30-year-old men and women, and previous research was in 50–69-year-old women [18], and 20–60 + year olds [17], where older adults and women are likely to be more health motivated than young adults and males [34].

Difficulties with blood flow and use of lancets align with the broader literature on the challenges of self-collection of samples using DBS cards in the general population [18]. The main challenge reported by participants was a lack of blood to fill the cards. It is possible that some participants felt discouraged when the process of self-sampling was more difficult than they originally anticipated, especially if their sample looked more like the “poor sample” than the “good sample” shown in the collection instructions. The present study was conducted during winter in Australia, where cold weather in the morning was specifically mentioned as a challenge by some participants and is likely to have indirectly contributed to the blood flow challenges reported by others. Nonetheless, some participants mentioned that following the instructions and collection tips helped facilitate the process, demonstrating the importance of the support provided. Difficulty with using the lancets was the most widely reported challenge after lack of blood flow. This is consistent with previous research, where 34% of written comments on difficulties with DBS cards related to the use, or lack of lancets [18]. In the present study, initially two lancets were provided in the kit (the standard number included by the pathology company), which the research team increased to three, then four, by the end of data collection in response to feedback from participants. Future research would benefit from pilot testing the collection process to ensure sufficient lancets are provided for the number of blood spots required and working with a consumer panel to refine procedures for achieving adequate samples in different population groups.

This research supports evidence of high prevalence of poor cardiometabolic health among young adults, particularly young males. The percentage at risk was greatest for total, HDL- and LDL-cholesterol, where 36%, 53%, and 44% of participants were at risk, respectively. This aligns with estimates from US national surveys that suggest up to 38% of young adults (18–29 years) had dyslipidemia in 2011–2018 [6], and that there has been a 4% relative increase in prevalence of diabetes per 2 year cycle in 18–44 year olds between 1999 and 2018 [35]. The percentage at risk was higher in males across all cardiometabolic measures, except for hsCRP, where prevalence was twice as high in females. This is consistent with literature on sex differences in cardiometabolic health [36,37] and may be partially explained by differences in weight status and other health behaviours between young males and females. This research supports the growing body of evidence that suggests worsening cardiometabolic health among young adults represents an important window of opportunity for early prevention of CVD [6,38,39]. Specifically, most research on CVD prevention, including risk prediction equations, focuses on adults 40 years or older and does not address the challenges of young people feeling disconnected from the healthcare system and not prioritising their long‐term health [39].

This study has implications for routine CVD risk assessment in young adults. The disconnect with healthcare professionals in this age group presents an opportunity for remote collection methods to remove barriers to accessing healthcare. Self-collection of samples is available in many countries for monitoring other aspects of health, such as sexual health [40], thus, extending this to other healthcare needs may represent an appropriate next step to improving participation in healthcare, particularly in underrepresented communities. Recommendations emerging from this research highlight strategies to optimise sample return and sample quality. Firstly, detailed step-by-step instructions and FAQs that include appropriate images and videos are essential for ensuring samples are collected adequately. Secondly, personalised support is required to assist participants with any difficulties they may experience, which may have implications for researcher burden. Future research should investigate low cost and innovative ways to support young adults who report any difficulties, such as online chat bots.

Strengths of this study include the large sample of young adults included from across Australia and the comparable numbers of males and females sampled. The collection of both quantitative and qualitative data enabled insights into barriers to the use of self-collected DBS cards. Several limitations are acknowledged. Convenience sampling resulted in higher proportions of participants with higher socio-economic position and data on ethnicity were not collected, thus feasibility and prevalence of poor cardiometabolic health cannot be inferred for lower socio-economic groups or the wider Australian population. Data on fasting was self-reported; thus, it was not possible to objectively discern whether cardiometabolic measures were reflective of fasting concentrations. The number and type of lancets provided may not have been adequate to collect sufficient blood for some participants. The example images of good-quality and poor-quality samples provided in the instructions may have deterred participants from completing and returning collection cards that were adequate for analysis of some measures. Thus, future research should ensure protocols are designed to include surplus lancets and appropriate images. As data on the feasibility of a 12-spot card compared to a 5-spot card was not collected, future research should determine whether a smaller card size would further improve feasibility. Lastly, to ensure feasibility and scalability of DBS screening, future research should investigate health and economic benefits of DBS kits for assessing metabolic risk compared to usual care.

This study shows the feasibility of self-administered DBS collection for cardiometabolic profile analysis in young adults aged 18–30 years. Step-by-step instructions and personalised and timely support are required to ensure sufficient quality samples are returned. Future research should ensure that kits contain surplus lancets and appropriate images to encourage participants to return their samples. The high proportions of young adults identified as at risk of poor cardiometabolic health highlight the need for routine CVD risk assessment to be extended to this age group.

## Supporting information

S1 Table STROBE-nut: An extension of the STROBE statement for nutritional epidemiology. (PDF)

S2 Table Response rate and adequacy of samples according to lancet number and type provided in DBS kit. Sarstedt, Sarstedt Safety lancet Extra Ø needle (85.1017); BD Microtainer, BD Microtainer contact-activated lancet (366578).
(PDF)

S1 Fig Printed booklet of instructions provided to participants in the dried blood spot card collection kits. Reprinted from NutriPATH under a CC BY license, with permission from NutriPATH, original copyright 2023.
(TIF)

S2 Fig Dried blood spot card cardiometabolic profile of young adults identified within optimal range (green), or outside optimal range, i.e., at risk (red) for a) total cholesterol (mg/dL), b) HDL-cholesterol (mg/dL), c) LDL-cholesterol (mg/dL), d) VLDL-cholesterol (mg/dL). (TIF)

S3 Fig Dried blood spot card cardiometabolic profile of young adults identified within optimal range (green) or outside optimal range, i.e., at risk (red) for a) high sensitivity c-reactive protein (hsCRP; mg/L), b) triglycerides (mg/dL), c) haemoglobin A1C (HbA1C; %) and d) insulin (µIU/mL). (TIF)

## Abbreviations:

CVD: Cardiovascular disease
DBS: Dried blood spot
HbA1C: Haemoglobin A1C
hsCRP: High sensitivity c-reactive protein
