# Supplementary material for: Feasibility of self-administered dried blood spot collection for cardiometabolic profile analysis in a population-based sample of young adults
Source: PLoS One. 2025 Oct 8;20(10):e0334023. doi: 10.1371/journal.pone.0334023 (PMC12507234; doi:10.1371/journal.pone.0334023)
Supplement: S2 Table — Sarstedt, Sarstedt Safety lancet Extra Ø needle (85.1017); BD Microtainer, BD Microtainer contact-activated lancet (366578). (PDF) [file pone.0334023.s002.pdf]

S2 Table. Response rate and adequacy of samples according to lancet number and type provided in DBS kit.

**Lancet type and number**	**Collection card returned** (n=366)	**Returned adequate sample**				
		**≥ 1 measure**	**≥ 2 measures**	**≥ 4 measures**	**≥ 6 measures**	**All 8 measures**
Sarstedt, n (%)						
Two	39 (68.4)	-	37 (94.9)	29 (74.4)	28 (71.8)	21 (53.8)
Three	12 (66.7)	-	-	10 (83.3)	9 (75.0)	8 (66.7)
Four	263 (73.5)	253 (96.2)	245 (93.2)	203 (77.2)	202 (76.8)	164 (62.4)
BD Microtainer, n (%)						
Four	52 (71.2)	51 (98.1)	50 (96.2)	44 (84.6)	44 (84.6)	42 (80.8)

Sarstedt, Sarstedt Safety lancet Extra Ø needle (85.1017); BD Microtainer, BD Microtainer contact-activated lancet (366578)
