# Supplementary figures and images for: Feasibility of self-administered dried blood spot collection for cardiometabolic profile analysis in a population-based sample of young adults
Source: PLoS One. 2025 Oct 8;20(10):e0334023. doi: 10.1371/journal.pone.0334023 (PMC12507234; doi:10.1371/journal.pone.0334023)

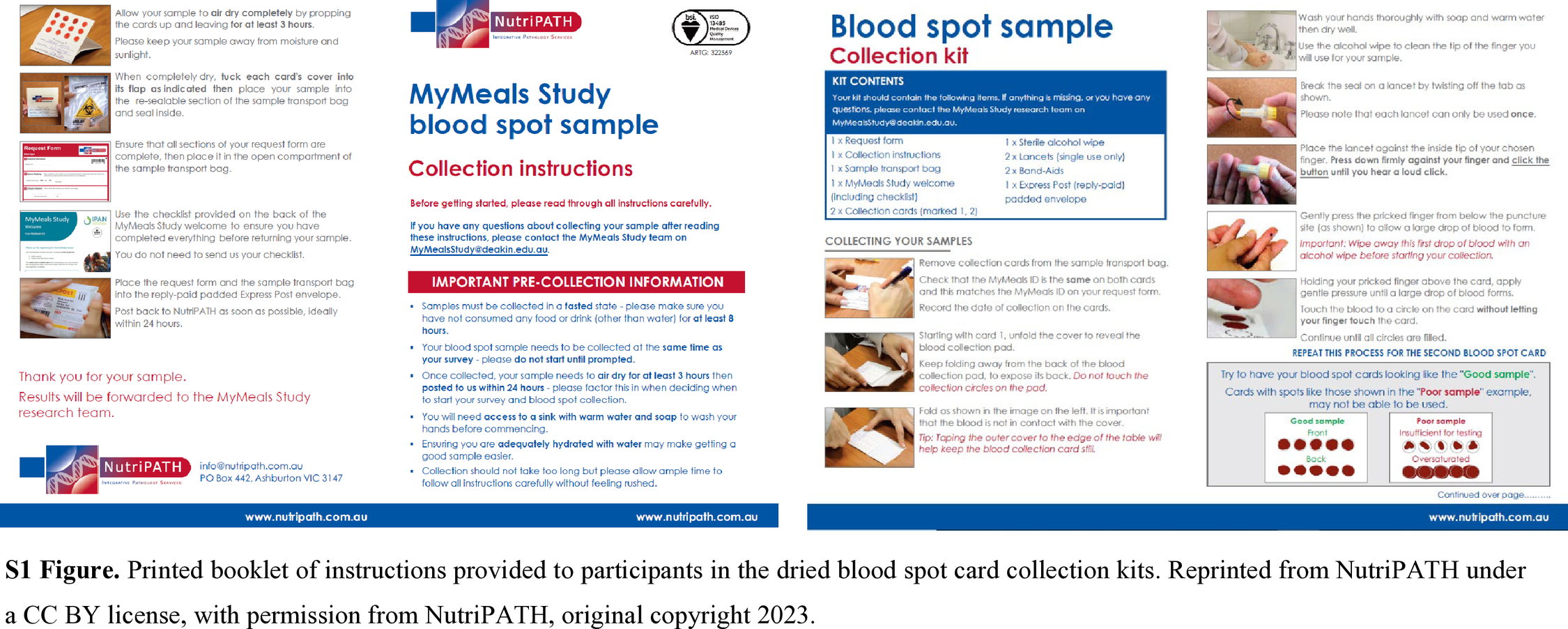

Supplement: S1 Fig — Reprinted from NutriPATH under a CC BY license, with permission from NutriPATH, original copyright 2023. (TIF) [file pone.0334023.s003.tif]

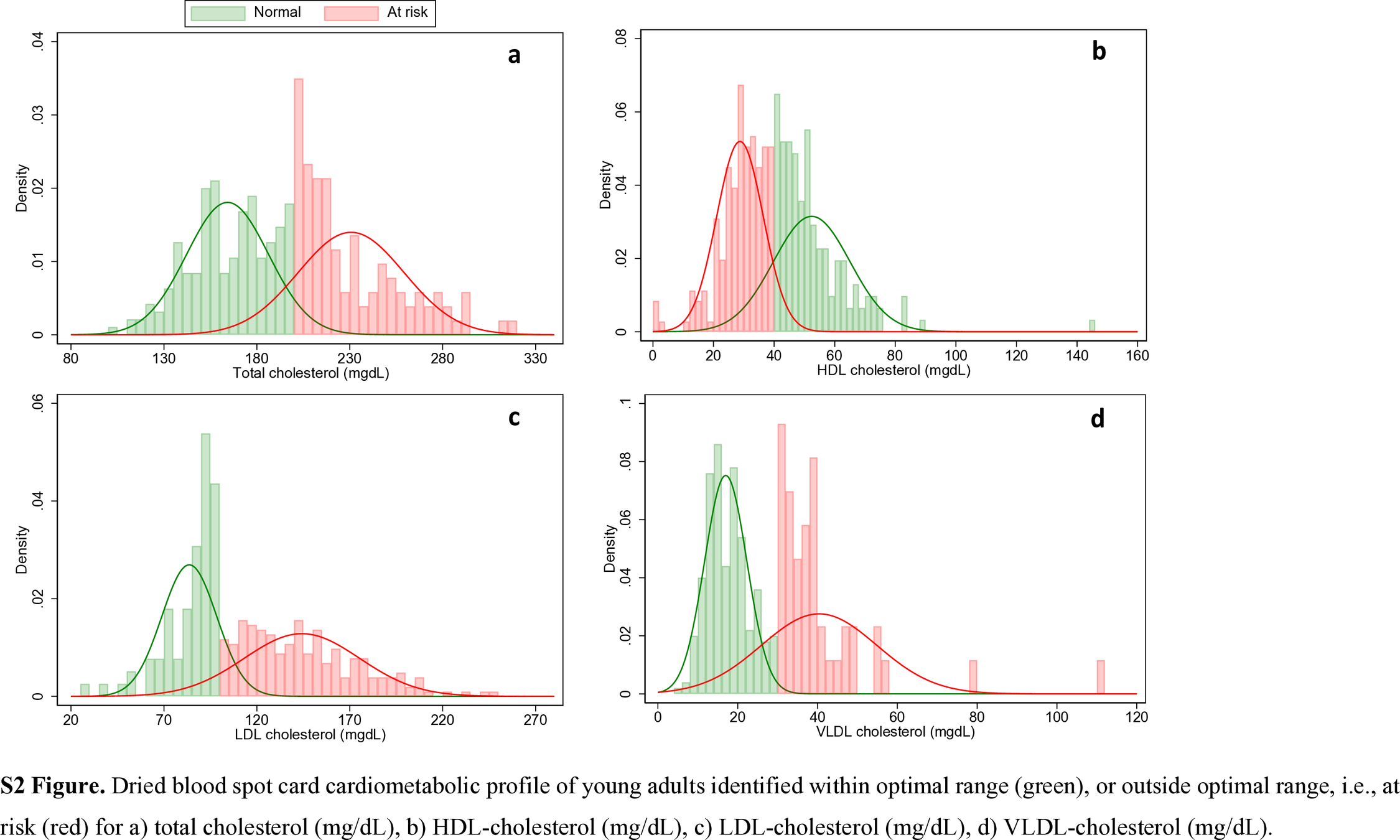

Supplement: S2 Fig — (TIF) [file pone.0334023.s004.tif]

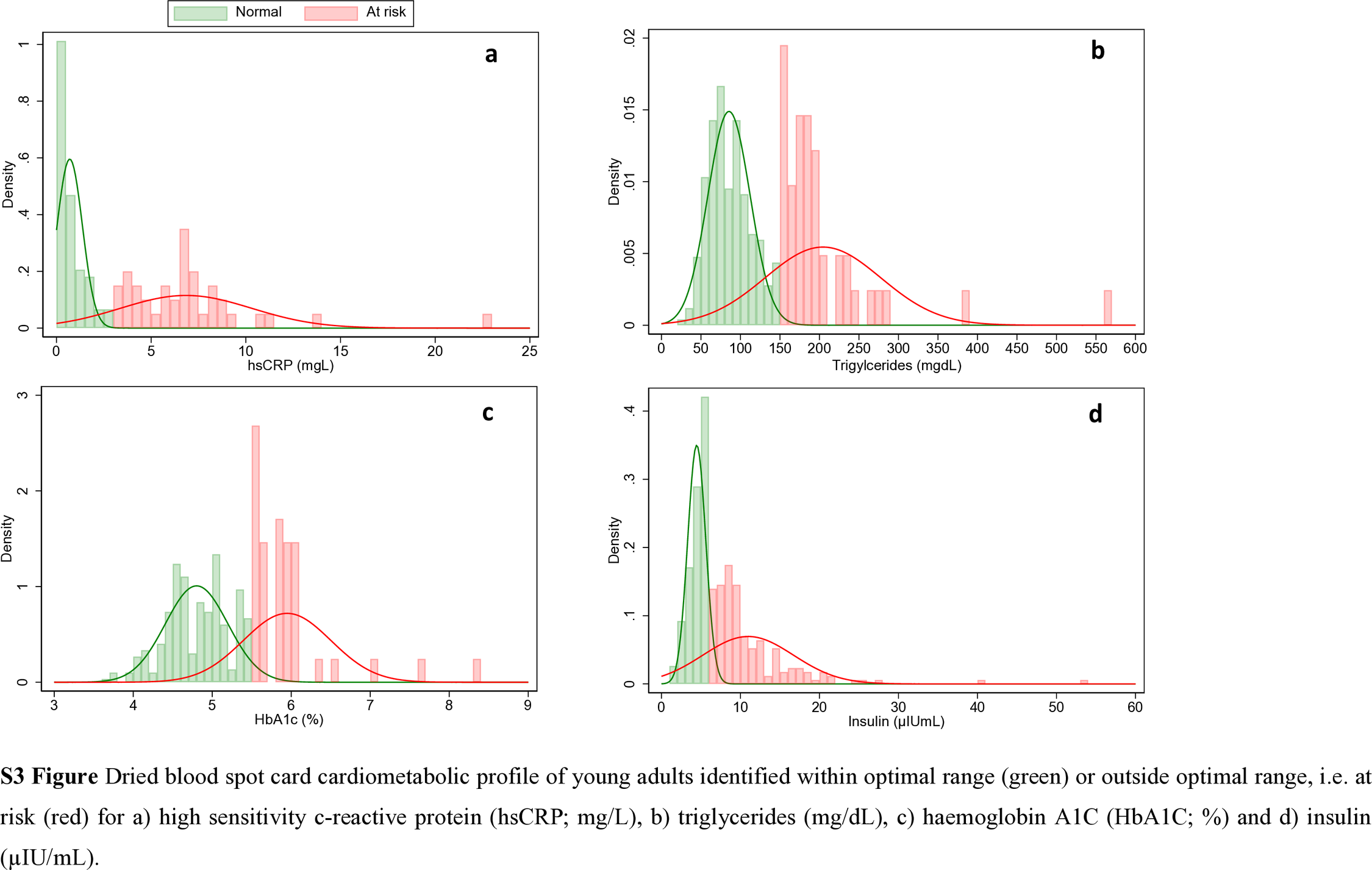

Supplement: S3 Fig — (TIF) [file pone.0334023.s005.tif]
